# Development of a national stroke audit in Ireland: scoping review protocol

**DOI:** 10.12688/hrbopenres.13244.1

**Published:** 2021-03-26

**Authors:** Carlos Bruen, Niamh A. Merriman, Paul J. Murphy, Joan McCormack, Eithne Sexton, Joseph Harbison, David Williams, Peter J. Kelly, Frances Horgan, Rónán Collins, Máirín Ní Bhreacáin, Elaine Byrne, John Thornton, Collette Tully, Anne Hickey

**Affiliations:** 1 Dept of Health Psychology, Royal College of Surgeons in Ireland, Dublin, Ireland; 2 Library Services, Royal College of Surgeons in Ireland, Dublin, Ireland; 3 National Office of Clinical Audit, Dublin, Ireland; 4 School of Medicine, Trinity College Dublin, Dublin, Ireland; 5 Dept of Geriatric and Stroke Medicine, St. James' Hospital, Dublin, Ireland; 6 Dept of Geriatric and Stroke Medicine, Beaumont Hospital, Dublin, Ireland; 7 Dept of Geriatric and Stroke Medicine, Royal College of Surgeons in Ireland, Dublin, Ireland; 8 Dept of Neurology, Mater Misericordiae University Hospital, Dublin, Ireland; 9 Neurovascular Clinical Science Unit, University College Dublin, Dublin, Ireland; 10 School of Physiotherapy, Royal College of Surgeons in Ireland, Dublin, Ireland; 11 Dept of Geriatric and Stroke Medicine, Tallaght University Hospital, Dublin, Ireland; 12 Patient Representative, Dublin, Ireland; 13 Institute of Leadership, Royal College of Surgeons in Ireland, Dublin, Ireland; 14 Dept. of Radiology, Beaumont Hospital, Dublin, Ireland

**Keywords:** Stroke, audit, registries, quality of care, outcome assessment, quality improvement

## Abstract

Introduction

Recent advances in stroke management and care have resulted in improved survival and outcomes. However, providing equitable access to acute care, rehabilitation and longer-term stroke care is challenging. Recent Irish evidence indicates variation in stroke outcomes across hospitals, and a need for continuous audit of stroke care to support quality improvement. The aim of this project is to develop a core minimum dataset for use in the new Irish National Audit of Stroke (INAS), which aims to improve the standard of stroke care in Ireland. This paper outlines the protocol for conducting a scoping review of international practice and guidelines in auditing acute and non-acute stroke care.

Objective

Identify data items that are currently collected by stroke audits internationally, and identify audit guidelines that exist for recommending inclusion of content in stroke audit datasets.

Methods and analysis

This scoping review will be conducted in accordance with the Preferred Reporting Items for Systematic Reviews extension for Scoping Reviews (PRISMA-ScR). We will search the following databases: Medline Ovid; Embase; CINAHL EBSCOHost. Grey literature will also be searched for relevant materials, as will relevant websites. Study selection and review will be carried out independently by two researchers, with discrepancies resolved by a third. Data charting and synthesis will involve sub-dividing relevant sources of evidence, and synthesising data into three categories: i) acute stroke care; ii) non-acute stroke care; and iii) audit data collection procedures and resourcing. Data will be charted using a standardised form specific to each category. Consultation with knowledge users will be conducted at all stages of the scoping review.

Discussion

This scoping review will contribute to a larger project aimed at developing an internationally benchmarked stroke audit tool that will be used prospectively to collect data on all stroke admissions in Ireland, encompassing both acute and non-acute data items.

## Introduction

Stroke is a major cause of death and disability globally ^1^. Each year in Ireland it is estimated that there are 7,700 new cases of stroke, and between 30,000 and 45,000 stroke survivors living either in the community or in nursing homes ^2^. In 2019, 12% of stroke patients in Ireland died in hospital - death from ischaemic stroke was 9% and from haemorrhagic stroke was 30%. Regarding ischaemic stroke, 72% of patients had disability on discharge while disability on discharge was 62% for haemorrhagic stroke ^3^. Key advances in acute stroke treatment in recent years have resulted in improved survival and patient outcomes, leading to more people in need of post-stroke care ^4^. Variability in the availability and quantity of post-stroke interventions and care can have a significant impact on outcomes for stroke patients, necessitating ongoing assessment of quality improvements in stroke management and quality of care.

In the context of a rapidly evolving evidence-base for stroke treatment and care, with considerable potential to improve outcomes for patients, there has been an emphasis on the clinical audit of stroke services to evaluate delivery of evidence-based best practice. Clinical audit is a “ *clinically-led quality improvement process that seeks to improve patient care and outcomes through systematic review of care against explicit criteria and acting to improve care when standards are not met.”* ^5^. Data on stroke care in hospitals has been collected since the 1950s - the first national stroke registry was established in Sweden in 1994 (Rik-Stroke) ^6^, with many countries developing similar registries since ^7^.

Stroke audit has made it possible to identify specific processes of care associated with better outcomes and improved service delivery in practice ^8^, e.g., early review of patients by a stroke consultant and early swallow assessment. It also makes it possible to identify the percentage of patients in receipt of care who meet defined standards and where there may be variation across hospitals. In Ireland, variation in stroke mortality across hospitals has been identified ^9^, highlighting the need for continual audit of clinical practice across sites.

### Development of a national stroke audit

The first national audit of stroke care in Ireland (2006–7) identified substantial deficits across key areas of stroke treatment and care, from emergency through to rehabilitation and secondary prevention ^10^. In 2010 the National Clinical Programme for Stroke was launched which, in partnership with the Economic and Social Research Institute (ESRI), developed the National Stroke Register in 2013 as a database for the systematic collection of stroke-related information. A second audit in 2015 showed encouraging improvements in many areas of stroke care, but also identified ongoing and substantial deficits ^11^. As part of its development, governance of the register was transferred to the National Office of Clinical Audit (NOCA) in 2019, and the register was renamed the Irish National Audit of Stroke (INAS).

A key next step in the development of this audit is to review the scope of the audit and to develop related core minimum datasets. Towards this, a research and knowledge user partnership was established in 2020 between key national stakeholders through the HRB-funded Applied Partnership Award (APA) “Maximising the Quality of Stroke Care in Ireland – Development of a National Stroke Audit”.

The output from this applied partnership will be an internationally benchmarked stroke audit tool that will be used prospectively to collect data on all stroke admissions in Ireland, from hyper-acute care through to rehabilitation and community care (also known as non-acute care). This dataset will be internationally benchmarked to maximise comparability with other countries to ensure that the audit is based on high quality reliable data that are maximally and nationally relevant. Furthermore, it will go beyond current practice internationally by including outcome data, both clinical and patient-reported, as well as including data on the rehabilitation and community care phases of treatment.

### Review objective

The objective of this scoping review is to identify data items that are currently collected by stroke audits internationally and to identify audit guidelines that exist for recommending content of stroke audit datasets. Through this, we will systematically review the literature detailing current international stroke audits to: i) identify a minimum dataset for acute stroke care; ii) identify a minimum dataset for non-acute stroke care; and iii) identify best practice for data collection procedures and resourcing for stroke audit. The current INAS dataset is also within the scope of the review and will be included in the charting and synthesis component, to ensure that the current Irish dataset is reviewed along with international practice.

## Methods & analysis

This scoping review will follow the six-stage format for scoping studies set out by Arksey & O’Malley ^12^ and advanced by Levac *et al.* ^13^, namely: 1. identifying the research question; 2. identifying relevant studies; 3. study selection; 4. charting the data; 5. collating, summarising and reporting the results; 6. consult with relevant stakeholders. The findings will be reported in accordance with the Joanna Briggs Institute (JBI) Preferred Reporting Items for Systematic reviews and Meta-Analysis extension for Scoping Reviews (PRISMA-ScR) reporting guidelines ^14^.

### Review question

The overarching review questions are defined as:

- What data items are currently collected by stroke audits internationally?
- What guidelines exist for the content of datasets for stroke audit?
- What procedures are used to collect data in stroke audits internationally, and how are these procedures resourced?

### Relevant studies

A search will be conducted for published literature on the following databases: Medline Ovid; Embase; CINAHL EBSCOHost. An information specialist (PM) has worked with the team in the development of the search strategy, and the search strategy was piloted and refined to check the appropriateness of keywords and databases prior to running the final search. Table 1 provides a sample Medline search string, which will be adapted for other databases. Grey literature will also be searched for relevant materials (e.g., using Google Scholar search engine), as will relevant websites, e.g., European Stroke Organisation. Research and knowledge user stakeholders within the APA partnership will be consulted to assist in the identification of relevant grey literature from their areas of knowledge and expertise. To ensure that sources are relevant and up-to-date, sources of evidence published since 2010 will be included.

**Table 1.** Sample Medline search string.

1	exp stroke/ OR stroke.mp. OR exp cerebral hemorrhage/ OR cerebral h*morrhage.mp. OR exp brain ischemia/ OR brain isch*mia.mp. OR exp Ischemic Attack, Transient/ OR isch*mic attack.mp.
2	((Stroke adj3 registry) OR (stroke adj3 registries) OR (National Stroke Registry) OR (National Stroke Register) OR (stroke adj3 audit$)).mp.
3	(quality register.mp. OR quality registry.mp. OR performance indicator*.mp. OR quality indicator*.mp.) AND stroke.mp.
4	((Audit adj2 guideline$) OR (registry adj2 guideline$)).mp.
5	2 OR 3 OR 4
6	1 AND 5
	limit 6 to yr="2010 – 2021"

All results will be imported into Endnote and duplicates removed. This search will be updated periodically after the project start date and a snowball search (backward and forward citation chasing) will be conducted to identify any further relevant documents citing or cited by the papers that are initially identified.

### Study selection

Following the PCC mnemonic (population, concept, and context) for scoping reviews ^15^, the inclusion criteria are as follows:

•   Population: The target population will be stroke. The definition of stroke used is broad, and includes transient ischaemic attack (TIA). Criteria will be revised as the review progresses (see below Consultation).

•   Concept and study type 1 - Audits: The review will initially follow inclusion criteria used in a review of national stroke registries ^16^, though adapted to also include stroke audit guidelines. The audit involves data collection for the purpose of monitoring and improving the quality of stroke care at country level. As with Cadilhac *et al.* ^16^, we consider a stroke registry to be at country level if it is reported as the accepted country-wide system for data collection; carries the name of a country; or is titled as ‘national’. We are guided by a general definition of a country as a United Nations (UN) member state or constituent country of a UN member state, and to include Taiwan despite its contentious political status. Audits at institutional or regional levels not part of a national network or informing national performance reviews will be excluded. In addition, the audit must be in operation in 2021. Criteria may be revised as the review progresses (see below Consultation).

•   Concept and study type 2 – Guidelines: Guidelines for data collection for the purpose of stroke audit or performance monitoring in relation to stroke care will be included.

•   Context: The review context is international, published since 2010 and in the English language, and focuses specifically on acute and non-acute stroke care settings.

Once an audit has been identified, there will be a targeted search process for specific documents of interest related to acute stroke care, non-acute stroke care, and audit data collection procedures and resourcing, e.g., through searches of the audit website. Eligible documentation will include published articles, reports, and methodology documents such as data dictionaries. 

Identified sources of evidence will undergo a two-level review process: a title and abstract review, and a full-text review. Two reviewers (NAM and CB) will independently apply the inclusion criteria to conduct a title and abstract review. Disagreements will be resolved by a third reviewer (AH), and through consensus-based discussion. For the full text review, two reviewers (NAM and CB) will independently review the documents to determine if they meet the inclusion criteria. Where an agreement cannot be reached, an independent third reviewer will be consulted (AH). Reporting of studies identified, included and excluded, will be documented through a PRISMA flow diagram, and details of excluded sources at full-text review stage will be appended to the review with reasons for their exclusion.

### Data charting

The search and selection process described above is designed to include national stroke audits and stroke audit guidelines of all types. The data charting and synthesis stages will involve sub-dividing relevant documents or sources of evidence, and synthesising data into three stand-alone categories: 1. acute stroke care; 2. non-acute stroke care; and 3. audit data collection procedures and resourcing. Data will be charted using a standardised form specific to each category, which will be piloted prior to data charting.

**Category 1: Acute stroke care audits & data**

The following information will be charted for Category 1:

Study type 1: Audits

- Audit characteristics and context: setting, years of operation, phases of care covered, number of sites, follow-up.
- Data items collected by the audit: for each item, we will record the question and potential response options, and the applicable/eligible patient population. Reporting methods will also be recorded, for example whether the indicator is risk-adjusted (e.g., hospital-specific mortality rates adjusted for case-mix). The rationale for inclusion of each item will also be identified, where possible.

Study type 2: Guidelines

- Context for each set of guidelines: setting, authors, development process.
- Recommended items: for each item, we will record the question and potential response options, and the applicable/eligible patient population. The rationale for inclusion of each item will also be identified, where possible.

**Category 2: Non-acute stroke care audits & data**

The specific information to be charted for Category 2 will include the same study types as those charted for acute care outlined above, and across the following categories: 1. audit characteristics and context; 2. data items collected by the audit; 3. context for each set of guidelines; and 4. guideline recommended items.

**Category 3: Stroke audit data collection procedures and resourcing**

Drawing on the same sources of evidence as above, data items for charting Category 3 will include:

•   Personnel – who collects and enters the audit data? How are they trained? What is routinely collected and collected specifically for audit purposes?

•   Infrastructure – IT systems, paper systems, data transfer procedures

•   Data quality – what checks or procedures are there for ensuring data quality?

•   Governance – what structures are in place in relation to consent?

•   Financing – is there financial support provided specifically to support audit data collection and related infrastructure?

### Synthesis

The research team (see Authors) will conduct an initial synthesis of findings, collating data items into three standalone categories: i) acute stroke care; ii) non-acute stroke care; and iii) audit data collection procedures and resourcing. This will result in an inventory of data items for both acute and non-acute stroke care, and will inform the production of an implementation strategy for data collection procedures for the national stroke audit in Ireland.

**Category 1: Acute stroke care audits & data**

The research team will conduct an initial synthesis of findings. Collation of data items included across audits will result in an inventory of acute care data items currently in use. Relevant sets of stroke audit guidelines will be combined to create a similar inventory.

**Category 2: Non-acute stroke care audits & data**

Data synthesis will follow the same procedure as for the acute care dataset, leading to an inventory of data items currently in use internationally and an inventory of recommended items for non-acute stroke care derived from stroke audit guidelines.

**Category 3: Stroke audit data collection procedures and resourcing**

Data synthesis will follow the same procedure as for both acute and non-acute stroke audit datasets. Following an initial synthesis of findings, scenarios will be produced outlining current international practice in relation to data collection procedures and resourcing for stroke audit.

### Consultation

This scoping review will contribute to a larger project aimed at developing a core minimum dataset for stroke care, and brings together academic researchers, knowledge users, and patient representatives. The National Office of Clinical Audit (NOCA) and the Quality Improvement Team within the Health Service Executive (HSE) in Ireland are the lead knowledge users, contributing equally to project development and financial support. A further key feature of this partnership involves regular and engaged consultation with the Governance Committee overseeing the Irish National Audit of Stroke (INAS), a multi-stakeholder group including clinical leads and experts, healthcare professionals and patient representatives responsible for making strategic decisions in relation to the audit. This includes regular consultation at all stages of the scoping review:

- **Search & study selection:** As they are identified, the results of the search and selection processes will be presented for discussion to the Governance Committee, providing an opportunity to revise the search strategy or inclusion criteria.
- **Data charting**: As the data are charted, results will be presented to the Governance Committee for discussion, potentially leading to revision of the charting framework to enable further searching to address gaps in the body of information.
- **Synthesis:** An inventory of data items will be presented to the Governance Committee to identify gaps and appropriateness for the Irish context. Based on this feedback, items will be prioritised that are supported by guideline recommendations, that occur across more than one audit, and that support the priorities identified by the Governance Committee.

This applied partnership also involves patient representation and key international collaborators who are involved in established national stroke audits. Patient representation includes co-applicants on the project design and grant proposal development, as well as ongoing consultation with co-applicants and patient representatives on the INAS Governance Committee. International collaborators include the UK Sentinel Stroke National Audit Programme (SSNAP), the Dutch Acute Stroke Audit (DASA) and the Canadian Registry of Stroke network. We will work with these patient representatives and international collaborators to develop and adapt the methodology for identifying and finalising the core minimum dataset required for a robust, benchmarked, comprehensive Irish National Audit of Stroke data collection tool.

## Discussion

### Implications

This scoping review will contribute to a larger project aimed at developing an internationally benchmarked stroke audit tool that will be used prospectively to collect data on all stroke admissions in Ireland, and importantly across the stroke care continuum to encompass both acute and non-acute data items. The resulting dataset will be the ‘gold standard’ to optimise comparability with other countries and ensure that high quality, maximally and nationally relevant and reliable data are included in the audit. Findings relating to resourcing of data collection procedures will inform future decision making relating to implementation of data collection strategies.

### Ethics and dissemination

The scoping review consists of reviewing and collecting data from publicly available materials, and therefore does not require ethics approval. The scoping review constitutes the first step in a multi-phased research project aimed at developing a minimum dataset for audit of stroke care. The results from this scoping review will guide and be combined with data from later phases of the research, including qualitative interviews and focus groups with stroke survivors, family members, healthcare professionals, and relevant research professionals and knowledge users (national and international). A Delphi Consensus Process will also be conducted towards the later phase of the project to inform the development of a minimum dataset. Ethics approval will be sought for these later stages of the research.

To facilitate knowledge translation, the research team will regularly consult with and disseminate findings to key stakeholders, knowledge users, and patient representatives. Findings will be disseminated through presentations and publications for both academic and non-academic audiences.

## Data availability

No data are associated with this article.
