# Supplementary material for: Development of a national stroke audit in Ireland: scoping review protocol
Source: HRB Open Res. 2021 Mar 26;4:31. [Version 1] doi: 10.12688/hrbopenres.13244.1 (PMC9607932; doi:10.12688/hrbopenres.13244.1)
Supplement: Peer review history

# Peer review: Reviewer response for version 1

**DOI:** 10.21956/hrbopenres.14407.r32449

**Published:** 2022-07-29

**Authors:** Urvashy Gopaul

**Affiliations:** 1 University Health Network - Toronto Rehabilitation Institute, Toronto, Canada; 2 University of Toronto, Toronto, Canada

This manuscript reports a scoping review protocol that focuses on international practice and guidelines in auditing acute and non-acute stroke care. The findings of this study are aimed towards the development of a core minimum dataset, for use in the new Irish National Audit of Stroke to improve the standard of stroke care in Ireland.

 The article is, overall, well-written. The topic and research question addressed in this study are relevant to improving stroke care for better outcomes in Ireland. The methods were clearly reported in sufficient details. This protocol provides a fair review and discussion.

 Some additional details/clarifications are required in some sections. I would like to recommend that this paper is accepted for indexing after the revisions have been done.

1. **Title: **The title gives the impression that the context of the scoping review is in Ireland. This title does not really match the specific questions of the scoping review. The context of the scoping review is international, even though the findings will later be used to determine audit items for Ireland. Title needs to be revised to reflect the broader context of the review.
2. **Abstract - 'Methods and analysis':** "Grey literature will also be searched for relevant materials, as will relevant websites". It is suggested to use an environmental scan as the method to identify materials as well as websites.
3. **Development of a national stroke audit: **"…but also identified ongoing and substantial deficits” - Can you list some of those deficits?
4. **Relevant studies:** 
  a) “Grey literature will also be searched for relevant materials (e.g., using Google Scholar search engine), as will relevant websites, e.g., European Stroke Organisation." This method of finding materials using search engines and websites is concurrent with an 'environmental scan'. The environmental scan could reinforce identification of materials that are not published in scientific journals. Please consider revising this section to reflect the best method to gather materials. Please also provide some more details about how materials will be selected from search engines such as Google.
  b) "All results will be imported into Endnote" - ****Please specify the software details such as company name, location and year version etc.
5. **Study selection: **"Population - The target population will be stroke". ****Please clarify if the stroke population will include adult stroke and well as the paediatric population - any age range?
6. **Consultation:** 
  a) It is recommended that stroke survivors are included in the consultation exercise too. The insight of stroke survivors could be very insightful in the rehabilitation and community care.
  b) "...patient representatives" - The choice of representative countries seems limited to few countries in Europe and Canada. Other countries that have conducted audits of acute services such as Sweden, Scotland, Australia etc. could also contribute to this exercise.
  c) To improve the flow of ideas and readability, it is suggested that this paragraph “This applied partnership also involves patient representation…collection tool.” is placed after the following sentence: "This includes regular consultation at all stages of the scoping review”.
7. **Discussion:** 
  a) It would be worth discussing some anticipated limitations of this study, e.g. the challenges with reference to obtaining materials about audits and data items from low and middle income countries which tend to be paper-based. How will you overcome this challenge? Maybe reach out to National stroke organizations or the Ministry of health?
  b) Also, language restriction to English could exclude access to valuable audits such as in China or France. It might be worth considering materials where translation in English is available.
8. **Table 1. Sample Medline search string:** Would it be worth adding the term ‘cerebrovascular accident*’? If not, why?

Is the study design appropriate for the research question?

Yes

Is the rationale for, and objectives of, the study clearly described?

Yes

Are sufficient details of the methods provided to allow replication by others?

Yes

Are the datasets clearly presented in a useable and accessible format?

Yes

Reviewer Expertise:

Stroke, rehabilitation, scoping reviews

I confirm that I have read this submission and believe that I have an appropriate level of expertise to confirm that it is of an acceptable scientific standard.

# Peer review: Reviewer response for version 1

**DOI:** 10.21956/hrbopenres.14407.r29385

**Published:** 2021-05-18

**Authors:** Terry Quinn

**Affiliations:** 1 Institute of Cardiovascular and Medical Sciences,, University of Glasgow, Glasgow, UK

The authors present the protocol for an evidence synthesis exercise that will collate information on various aspects of international stroke audit activity and method.

 This is a well written protocol for an important piece of work that will inform subsequent national stroke audits.

 I have only minor comments and suggestions to improve clarity:

- The title doesn’t make it clear what is being scoped in this review – could the detail of the scoping be added to the title?
- The method is clear.
- The review methods are appropriate, but I wonder if the data sources are too focused. The method described is reliant on details of national audits being published in traditional scientific journals. This may not always be the case. Combining the electronic literature search with purposive searching, reaching out to national stroke societies will ensure that no relevant audits are missed.
- There is also the danger that the published content accessed during the search is out of date. For the two audits that I know best, Scottish Stroke Care Audit (SSCA) and Sentinel Stroke National Audit Program (SSNAP), the data items are being revised at present and content described in older publications will be obsolete. Again, reaching out to the leads of each audit identified will help ensure that the data are up to date and reflect current audit practice.
- Something to reflect on in the dissemination and stake holder engagement events, is the differential approach that various national audits take where there is established best practice in stroke care but supporting evidence is weak or non-existent. This is particularly relevant to many aspects of post-acute stroke care.
- I was pleased to see detailed definitions of the core concepts in the review. I wonder if even more detailed operationalisation is needed.
  For example, the current definition of a national audit may exclude the AHA ‘Get With The Guidelines’ program and the pan-European Safe Implementation of Treatment in Stroke (SITS) – is this the intention?  
  The ‘non-acute’ setting also needs further detail in the definition. Would this include primary care (eg the NHS Quality Outcome Framework)
- I was pleased to see plans for active engagement with stakeholders, including national stroke audit organisations. Why is this limited to NHS England, Netherlands and Canada? I am fairly sure that the Scottish Stroke Care Audit would also be keen to contribute and share knowledge and experience.

Some minor points:

- The description of data sources as written in the manuscript seems to conflate databases eg EMBASE with the information service provider eg EBSCOhost.
- How will Google Scholar be used? Common practice is to limit to first hundred titles returned ‘Relevant websites’ will be accessed, could these be listed ?

Is the study design appropriate for the research question?

Yes

Is the rationale for, and objectives of, the study clearly described?

Yes

Are sufficient details of the methods provided to allow replication by others?

Yes

Are the datasets clearly presented in a useable and accessible format?

Yes

Reviewer Expertise:

Stroke, evidence synthesis, guidelines

I confirm that I have read this submission and believe that I have an appropriate level of expertise to confirm that it is of an acceptable scientific standard.
